# Palliative care needs of patients living with end-stage kidney disease not treated with renal replacement therapy: An exploratory qualitative study from Blantyre, Malawi

**DOI:** 10.4102/phcfm.v9i1.1376

**Published:** 2017-05-29

**Authors:** Maya J. Bates, Alex Chitani, Gavin Dreyer

**Affiliations:** 1 College of Medicine, Blantyre, Malawi; 2 Ministry of Health, Blantyre, Malawi; 3 Barts Health NHS Trust, London, United Kingdom

## Abstract

**Background:**

The burden of end-stage kidney disease (ESKD) in sub-Saharan Africa is increasing rapidly but the palliative care needs of patients living with ESKD are not well described. Resource limitations at both health system and patient level act as major barriers to patients receiving renal replacement therapy (RRT) in the form of dialysis. We undertook an exploratory qualitative study to describe the palliative care needs of patients with ESKD who were not receiving RRT, at a government teaching hospital in Blantyre, Malawi.

**Methods:**

A qualitative, explorative and descriptive design was used. Study participants were adults aged > 18 years with an estimated glomerular filtration rate < 15 ml/min on two separate occasions, three months apart, who either chose not to have or were not deemed suitable for RRT. Data were collected by means of semi-structured interviews.

**Results:**

In October and November 2013, interviews were conducted with 10 adults (7 women with median age of 60.5 years). All were hypertensive and four were on treatment for HIV. Four themes emerged from the data: changes in functional status because of physical symptoms, financial challenges impacting hospital care, loss of role within the family and the importance of spiritual and cultural beliefs.

**Conclusion:**

This study reports on four thematic areas which warrant further quantitative and qualitative studies both in Malawi and other low-resource settings, where a growing number of patients with ESKD unable to access RRT will require palliative care in the coming years.

## Introduction

The small amount of research on end-stage kidney disease (ESKD) published from within Africa focuses on the epidemiology of renal replacement therapy (RRT) provision and its costs, rather than quality of life.^1^ For those few adults who receive dialysis outcomes are generally poor and default rates high.^2^ Chronic kidney disease (CKD) is at least three to four times more prevalent in Africa than in developed countries.^3^ The global burden of CKD is expected to increase rapidly and particularly in sub-Saharan Africa (SSA) as a result of the rising prevalence of risk factors such as acute kidney injury, diabetes mellitus and hypertension (HTN).^4^ In this setting, many people present with their illness at a very advanced stage.^1^ RRT with dialysis forms a small but challenging part of global nephrology, with the extremely high cost of this intervention leading to limited access for patients with ESKD. Even in settings where RRT is readily available, conservative management alternatives are being increasingly considered for certain groups (including the elderly and those with multiple comorbidities) for whom RRT holds little or no improvement in the quality of life.^5^ Palliative care services in the continent have expanded over the last 10 years, though the focus has been largely on HIV and cancer-related diagnoses.

Malawi is a small, densely populated country in southern Africa, where over half of the population of almost 16 million people live below the poverty line. Life expectancy is 55 years and adult prevalence of HIV infection is 10.8% (UNICEF data, Malawi 2013).^6^ Current treatment options available for patients with ESKD are haemodialysis and palliative care. Chronic peritoneal dialysis is not available in Malawi. Haemodialysis has been available free at the point of access since 1998 at Kamuzu Central Hospital in Lilongwe, where there is limited provision of palliative care. Queen Elizabeth Central Hospital (QECH) in Blantyre is the largest public hospital in the country with over 1200 beds. Hospital-based palliative care for adult patients started in 2003,^7^ and a four station haemodialysis unit has been operational since 2011. Chronic haemodialysis with supporting renal clinics is available for a limited number of patients at these two tertiary centres. Decisions on who should receive dialysis were made on a case-by-case basis. Priority was given to patients who met certain criteria: age < 50, hepatitis B and HIV negative, body mass index < 30 and no serious comorbidities.

There is a dearth of literature concerning quality of life outcomes of patients with ESKD not receiving RRT from an African setting. That which is available is for patients treated with RRT, including a recent quantitative study at our hospital examining the quality of life of patients with ESKD treated with RRT using structured questionnaires.^8^ Small et al.^9^ explored the quality of life and palliative care needs of patients receiving haemodialysis for ESKD in Namibia using a qualitative approach. The issues which emerged from the interviews were loss of independence and spontaneous activities, and strain on relationships and feelings of physiological changes and weakness.

For those not receiving RRT, a recent systematic review of the literature highlighted the heavy symptom burden experienced by patients and the importance of palliative care.^5^ In contrast to high-resource settings, such ‘conservative management’ of patients with ESKD in low-resource settings is largely dictated by health system constraints^1^ rather than patients’ choice. The needs of patients with ESKD not receiving RRT in SSA are not currently represented in the literature. Recognition of the needs of this patient group by the specialist renal physician (G.D.) led to the collaboration between renal and palliative care specialist teams to undertake this study. Because of the paucity of both renal and palliative care services across much of SSA,^10^ there are currently few places which combine the necessary expertise to undertake a study of this kind. As this patient population is expected to grow in the coming years, we decided to undertake an exploratory qualitative study to describe their palliative care needs in order to inform service providers and identify areas for future study.

## Methods

This study aimed to describe the palliative care needs of patients with ESKD not treated with RRT at QECH, Blantyre, Malawi.

Purposive sampling was used to select eligible patients from amongst those under review at the weekly renal specialist clinic at our hospital. Study participants were adults aged > 18 years with a diagnosis of ESKD, as determined by an estimated glomerular filtration rate of < 15 ml/min (based on the modification of diet in renal disease equation^11^ on two separate occasions, three months apart with no reversible element to their kidney disease. Prior to the study recruitment, they had either chosen not to have, or were assessed by a renal specialist physician (G.D.) as being unsuitable for, RRT and were being managed with medical therapy only.

Data were collected by means of semi-structured interviews that were conducted using an interview guide (Box 1). The interview guide was piloted and adjusted following observations made during the pilot. Sample size was determined with reference to thematic saturation, though the timeframe and the funding available for data collection were also limiting factors. Interviews were conducted by an experienced Malawian clinician (A.C.), not previously known to participants, fluent in Chichewa (national language) and English, who approached the participants face-to-face during the renal clinic. Written, informed consent was obtained from all the patients who were then interviewed individually away from the clinic setting. Interviews lasted for about 45–60 min. Interviews were conducted in the language of preference of the participant and tape-recorded. The recordings were transcribed verbatim and, where necessary, interviews were translated from local language (Chichewa) to English. Field notes were made recording observations made by the researcher during the process of interviewing the participants.

BOX 1 Key areas of the interview guide.

**Table:** 

General life history (Tell me about your life at the moment?)
Symptoms attributed to kidney disease (What do you think is the cause of your physical problems?)
Ways of coping (What keeps you going?)
Life priorities (What are your main concerns right now?)
Disease understanding (How do you see the future?)

Thematic content analysis was used to identify codes which were identified and gathered into named themes. Two members of the research team (A.C. and J.B.) separately read and reread the interview transcripts to familiarise themselves with the data. Initial manual coding was done by A.C. and then separately by J.B. After discussion, final themes and sub-themes derived from the collected data were agreed upon.

### Ethical consideration

Ethical approval to conduct the study was obtained from the College of Medicine Research Ethics Committee (protocol number P.03/13/08). Written consent was provided by all study participants prior to recruitment into the study. Completed consent forms are available on request. The English translation transcriptions of tape-recorded data are available on request from A.C.

## Results

Ten adult patients with ESKD were interviewed; two other patients who were approached for recruitment declined because of reported time constraints during their clinic visit (they lived far from the hospital). The mean age of participants was 58 years (median 60.5 years, range 34–68). Seven were female. All interviewees had a history of HTN, two were diabetic and four were HIV positive (all of these patients were receiving highly active antiretroviral therapy [HAART]). The clinical and demographic data of study participants are summarised in Table 1.

**TABLE 1:** Clinical and demographic details of interviewees.

Patient	Age (years)	Gender	Marital status	HIV status	Risk factors	Occupation	Comorbidity
P01	68	F	Married	Negative	HTN	Retired businessman	Stroke
P02	68	M	Divorced	Negative	HTN	Retired farmer	CCF
P03	34	F	Married (second husband)	Positive	HTN	Retired, business and farming	
P04	60	F	Widow	Negative	HTN	Retired, small-scale business	-
P05	63	M	Married	Negative	HTN	Retired builder	Stroke
P06	54	F	Divorced	Negative	Diabetic HTN	Retired businessman	Stroke Diabetic retinopathy
P07	61	F	Widow	Positive	Diabetic HTN	Retired nurse	-
P08	57	F	Married	Negative	HTN	Retired civil servant	-
P09	64	M	Married	Positive	HTN	Retired officer	-
P10	49	F	Widow	Positive	HTN	Farmer	CCF, unspecified gynaecological malignancy

CCF, congestive cardiac failure; HTN, hypertension.

Four major themes emerged from the interviews. These were as follows: changes in functional status, financial challenges impacting hospital care, loss of role within the family and the importance of spiritual and cultural beliefs (Table 2).

**TABLE 2:** Themes and sub-themes developed in data analysis.

Theme 1: Changes in functional status	Theme 2: Financial challenges impacting hospital care	Theme 3: Loss of role within the family	Theme 4: Importance of spiritual and cultural beliefs
Pain and insomnia	Transport and missing follow-ups	Loss of role as carer	Hope
Breathlessness	Drugs out of stock	Loss of role as sexual partner	Acceptance of the condition and future
Nausea and vomiting	Drugs expensive at private pharmacies	Loss of role as breadwinner	Belief in witchcraft
Depression	-	-	-

### Theme 1: Changes in functional status

Patients reported that their health changed as a result of ESKD.

‘My life has been very difficult, I can’t eat properly, I don’t have strength and to go to the toilet to urinate its very painful because I have to take drugs for me to go and urinate, … feeling nausea, feeling as even I want to vomit all the time.’ (P06, female, 54 years old)

‘To reach the place where I can get a minibus is very difficult; I have to walk very slowly and have to rest because I feel very tired. And then I reach the hospital very late while also the clinic has already finished.’ (P02, male, 68 years old)

#### Pain and insomnia

A number of patients interviewed complained of uncontrolled pain:

‘My son has been taking me to different hospitals so that maybe we can find suitable medication for my pain of the legs but I have been given the same medications.’ (P01, female, 68 years old)

Some patients experienced insomnia as a result of uncontrolled pain.

‘I am having pain in my legs and I cannot sleep at night, I even wake up at night, sometimes numbness of legs.’ (P02, male, 68 years old)

#### Breathlessness

All those interviewed experienced shortness of breath at some stage which affected their function.

‘When I walk a small distance I have to rest because of breathlessness.’ (P01, female 68 years old)

#### Nausea and vomiting

Nausea and vomiting were common complaints, either from the disease or linked to medication that had been prescribed.

‘I feel nausea all the time and sometimes I vomit.’ (P06, female, 54 years old)

‘The doctor gave the prescription to buy the drugs. After taking those I was vomiting continuously I had to come back to the hospital and the doctor stopped the drugs.’ (P10, female, 49 years old)

#### Depression

Respondents spoke of the psychological impact of their diagnosis; two patients cried during the interviews.

‘I was in denial and depressed at that time, I stayed home without going to work and my blood pressure was very high, being a nurse, you know these conditions and their outcomes … I couldn’t take it.’ (P07, female, 61 years old)

Another patient commented:

‘I was very devastated the time I was told that my kidneys are damaged and will not work normally.’ (P08, female, 57 years old)

### Theme 2: Financial challenges and impact on care

Respondents spoke of numerous ways that their illness brought about financial difficulties, which in turn affected their care.

#### Transport and missing follow-up appointments

‘It is very difficult for me to find money for transport to come to the hospital because I came with my guardian and we use MK3700.00 (approximately $8) per visit which is also difficult because now I cannot work.’ (P02, male, 68 years old)

#### Payment for medications

‘Every time when you go to get some medication in our government pharmacy, they [*medications prescribed*] are not available. They [*health workers*] tell us to go and buy. The medication is very expensive [*$5*] which I cannot afford. I just go back home without the medication.’ (P08, female, 57 years old)

### Theme 3: Loss of role within the family

#### Loss of role as a carer

Respondents spoke of the changing role as their children care for them.

‘My children usually find temporary jobs which, when they find a little something, they help me. Apart from my children there’s no one who helps me … even the church does not help.’ (P02, male, 68 years old)

#### Loss of role as sexual partner

One respondent discussed the impact of illness on his sexuality.

‘This disease has really affected my family life because I do not have sexual feelings these days. I don’t have strength, I feel weak and breathless.’ (P02, male, 68 years old)

Another mentioned loss of libido as a concern.

‘Because I have been having pain with my legs and all over my body this has also contributed for me not having feelings for sex with my wife.’ (P05, male, 63 years old)

#### Loss of role as a breadwinner

‘I used to do farming but now I cannot because I do not have strength.’ (PO2, male, 68 years old)

‘I was doing a small business like going to Mangochi to buy fish and sell them here in Blantyre and baking donuts. It’s very difficult to find help.’ (P04, female, 60 years old)

One respondent reported the added strain on his marriage in this way:

‘[*I*] am disappointed because we don’t ‘agree to disagree’ in my family as husband and wife.’ (P05, male, 63 years old)

When asked more, he mentioned that the difficulties were occurring because he was unable to work and money was short.

‘Because I don’t have money to feed my family and it’s difficult to find it. You know these days for everything to work you must have money.’ (P05, male, 63 years old)

Children were also affected by financial challenges as payment of school fees became difficult as a result of ill health.

‘I find it difficult to pay school fees for my children since I can no longer do my business.’ (P05, male, 63 years old)

### Theme 4: Importance of spiritual and cultural beliefs

Spiritual and cultural beliefs, including faith and prayer, were reported by respondents as ways in which they keep going, despite the limitations of their illness. All patients interviewed expressed the importance of faith.

#### Hope

Faith (in God) gave respondents hope.

‘Prayer is powerful because in whatever I have been through … I know with God everything is possible. With the problems I have been through they make to be closer to him.’ (P08, female, 57 years old)

However, some patients expressed less hope for their future.

‘My future is uncertain because I see that my life is deteriorating.’ (P07, female, 61 years old)

#### Acceptance of condition and future

Spiritual and cultural beliefs had a role in respondents’ acceptance of their condition.

‘I know it [*kidney disease*] is the will of God.’ (P02, male, 68 years old)

‘Doctors play their own part and have their limits while God gives them wisdom and continue from where they have failed.’ (P08, female, 57 years old)

One respondent expressed his beliefs about the cause of ESKD according to local cultural belief of witchcraft.

‘I thought I was bewitched and we nearly fought at home with knives because my mother was convinced that I was bewitched. I went to a traditional doctor but I was not improving. Now I am fine I thank the hospital. I could have died.’ (P10, female, 49 years old)

## Discussion

We present our findings from an exploratory qualitative study, which describes the palliative care needs of patients living with ESKD not treated with RRT at QECH in Blantyre, Malawi. We believe this is the first study of its kind from within SSA. Use of a qualitative approach enables data collection and analysis from a person-centred perspective, which is the focus of palliative care.

Our findings reflect the context in which the patients and their families are living with a serious terminal diagnosis, with an emphasis on financial concerns, sexuality and spiritual and cultural beliefs. Themes reflect concerns which go beyond themselves to affect the lives of their carers and dependents (though none of these groups was included within the scope of this study).

The first theme highlights the heavy burden of physical symptoms experienced in this patient group, resulting in changes in their functional status. Reported symptoms are similar to those which have been described through larger quantitative studies of patients with ESKD in high-resource settings.^12^ It was outside the scope of this study to determine the precise symptom burden or describe the aetiology of ESKD, but it was noted that underlying comorbidities were common as found in high-resource settings.^13^ All respondents were hypertensive and four were infected with HIV requiring HAART. One had advanced gynaecological malignancy. It is unclear how much these comorbid conditions influenced the reporting of symptoms. However, all patients in the study had advanced kidney disease to a point where we consider that the medical and psychological aspects of ESKD itself were the major influences on symptoms. The age of patients with ESKD in our study (median age 60.5 years) is not dissimilar to the age of patients requiring haemodialysis in other settings,^14^ but is much lower than the age of patients being considered for conservative management in high-resource settings.^5^ Patients reported financial challenges impacting hospital care in our study. Sub-themes included concerns about costs of transport to hospital for follow-up and payment for (relatively inexpensive) medications which were out of stock in the government pharmacy or expensive when patients were forced to source these themselves (through private pharmacies). These findings are similar to reports from patients with a variety of conditions receiving palliative and end of life care in SSA,^15,16^ where health systems are often weak and issues such as drug stock-outs are fairly common. Our study was conducted by recruiting patients from a non-fee-paying public hospital specialist renal clinic to which, apart from the cost of travel to the clinic, there were no obvious financial barriers. Despite this, out-of-pocket expenses were of considerable concern. Luyckx et al.^17^ mentioned the potential for ‘catastrophic payments’ for patients with kidney disease in SSA in their commentary on priorities for treatment in the region, while a recent Lancet editorial highlights the results of a systematic review which reports that of the tiny minority of patients in SSA who ever received dialysis, most default on treatment as a result of the inability to pay.^2,18^ Our study used a qualitative approach and was therefore not designed to quantify the actual burden of either direct or indirect costs experienced at household level by patients and families living with ESKD; more detailed work in this area would be valuable in future.

Our third identified theme was that of loss of role. Patients expressed this loss in terms of their expected role as carers, sexual partners and breadwinners. The average age of respondents was 58 years (median 60.5, range 34–68 years). Many respondents commented on their loss of role as breadwinners despite the fact that all but one of them described themselves as ‘retired’. This perhaps reflects contextual issues such as relatively early age of retirement (minimum age of retirement is 50 years in Malawi) as well as the frequent necessity for older people (particularly men) to support their dependents in the absence of any form of state provision of welfare.^19^

It is interesting to note the comments made by patients with ESKD about their loss of role as sexual partners; this is in keeping with research amongst cancer patients in North America, which concluded that *sexuality continues to be important even at the end of life*.^20^ More work is needed to explore the importance of this loss of role as a sexual partner, how it may affect quality of life and culturally appropriate strategies to alleviate suffering.

The final theme highlights the importance of spiritual and cultural beliefs in maintaining hope as well as with reference to understanding and acceptance of their terminal diagnosis. Assessment of spiritual needs is an important component of palliative care as defined by the World Health Organization, and recommendations have been produced for spiritual care in palliative care in Africa.^21^ In our study, the respondents pointed to the importance of their belief in God in providing hope and acceptance of their disease. Disease understanding for one patient (respondent 10) incorporated beliefs about witchcraft. Such beliefs are widely encountered in clinical practice in Malawi and have been documented as influencing a number of disease states.^22,23^ Studies conducted in other African settings exploring beliefs around chronic disease causation have reported similar findings.^24,25^ The importance and impact of spiritual and cultural beliefs (incorporating local health beliefs) in the understanding and acceptance of advanced disease and the meaning of hope would provide interesting avenues for further qualitative study in Malawi and similar settings.

## Conclusions

Improving access to affordable palliative care is an important priority in response to the challenges posed by the global rise in ESKD. This is not currently reflected in the emerging body of literature about the global response to kidney disease (The Lancet Global).^18^ This exploratory qualitative study provides insight into the palliative care needs of patients with ESKD not treated with RRT from the setting of a central teaching hospital in Blantyre, Malawi, where they are managed through collaboration between renal and palliative care services.

Our findings concur with quantitative studies from high-resource settings where patients expressed concerns about symptom burden limiting their functional ability. Other themes identified in our study reflect some of the contextual realities of a low-resource environment. The diagnosis of ESKD presents extreme financial challenges, which impacts on routine visits to hospital and purchase of essential medication. Loss of roles within the family – as carer, sexual partner and breadwinner – was of importance to those interviewed. Spiritual and cultural beliefs were a source of hope as well as framing understanding and acceptance of disease. Such beliefs also led one respondent to report that the disease was caused by witchcraft.

This small, exploratory, qualitative study reports on four thematic areas which warrant further quantitative and qualitative studies both in Malawi and other low-resource settings, where a growing number of patients with ESKD managed without RRT require care in the coming years. For the majority of patients who are diagnosed and reviewed by renal services without access to RRT or formal palliative care provision, clinicians and nurses should adopt some simple tools, using a symptom-based approach. By asking the patient *what is your main concern?* and *do you have any questions?*, patient-centred priorities can be identified and supported to optimise the quality of life up to and beyond the time of death.
